# Recurrent giant cystic lymphangioma of peritoneum: a case report and literature review

**DOI:** 10.3389/fonc.2024.1449870

**Published:** 2024-10-01

**Authors:** Hongyan Lei, Jinxin Sun, Yongzhou Wang, Tao Ye

**Affiliations:** Department of Gynecology, The Affiliated Traditional Chinese Medicine Hospital, Southwest Medical University, Luzhou, China

**Keywords:** cystic lymphangioma, surgical resection, case report, recurrence, treatment

## Abstract

Cystic lymphangioma (CL) is a rare benign tumor of the lymphatic system, most commonly found in the neck, head, and armpits. The incidence of lymphangiomas in the abdominal cavity is less than 5%, and the incidence of retroperitoneal tumors is even lower. We report here a case of recurrent giant CL that recurred 20 years after the first complete surgical resection. Pelvic MRI at 6 months follow-up after the second complete surgical resection indicated a recurrence of the tumor. The main purpose of this article is to explore the treatment and follow-up strategies for recurrent lymphangioma.

## Introduction

CL is a cyst formed by congenital lymphatic malformation, which can occur in anywhere in the body (1). It originates from lymphatic endothelial cells and is believed to be due to the dilation or abnormal development of lymphatic vessels (2). CL is more common in the neck or armpit, accounting for only 5% of lesions in the abdominal lymphatic vessels, and retroperitoneal lymphangioma is even rarer (3). It can occur at any age, but is more common in children (4). Histologically, it consists of an enlarged cystic space containing a proteinaceous fluid with thin septa lined by endothelial cells[ (5). Preoperative diagnosis is challenging, with a wide range of differential diagnoses. The diagnosis of CL mainly relies on imaging examinations such as ultrasound (US), computed tomography (CT), or magnetic resonance imaging (MRI), and confirmation through histology (6). In cases where surgical resection is possible, complete excision should be pursued. The risk of recurrence depends mainly on the status of the surgical margins (7). Here, we report a case of a female with a large CLin the abdomen that underwent excision, considering the possibility of recurrence due to its large size and unclear boundaries around the pelvic region, and review the literature on this topic.

## Case description

The patient, a 47-year-old female, visited our hospital on January 9, 2023. One month prior, she began experiencing abdominal pain, bloating, and constipation of unknown cause. Ultrasound of the upper abdomen and gynecological examination revealed a cystic-solid mass in the abdomen and pelvis (a mixed echo mass was detected in the abdomen and pelvis, with numerous reticular septa, too large to measure: extending from above the xiphoid process to the pelvis, with punctate blood flow signals within). The patient was admitted for further diagnostic evaluation and surgical intervention.

**Figure 1:**
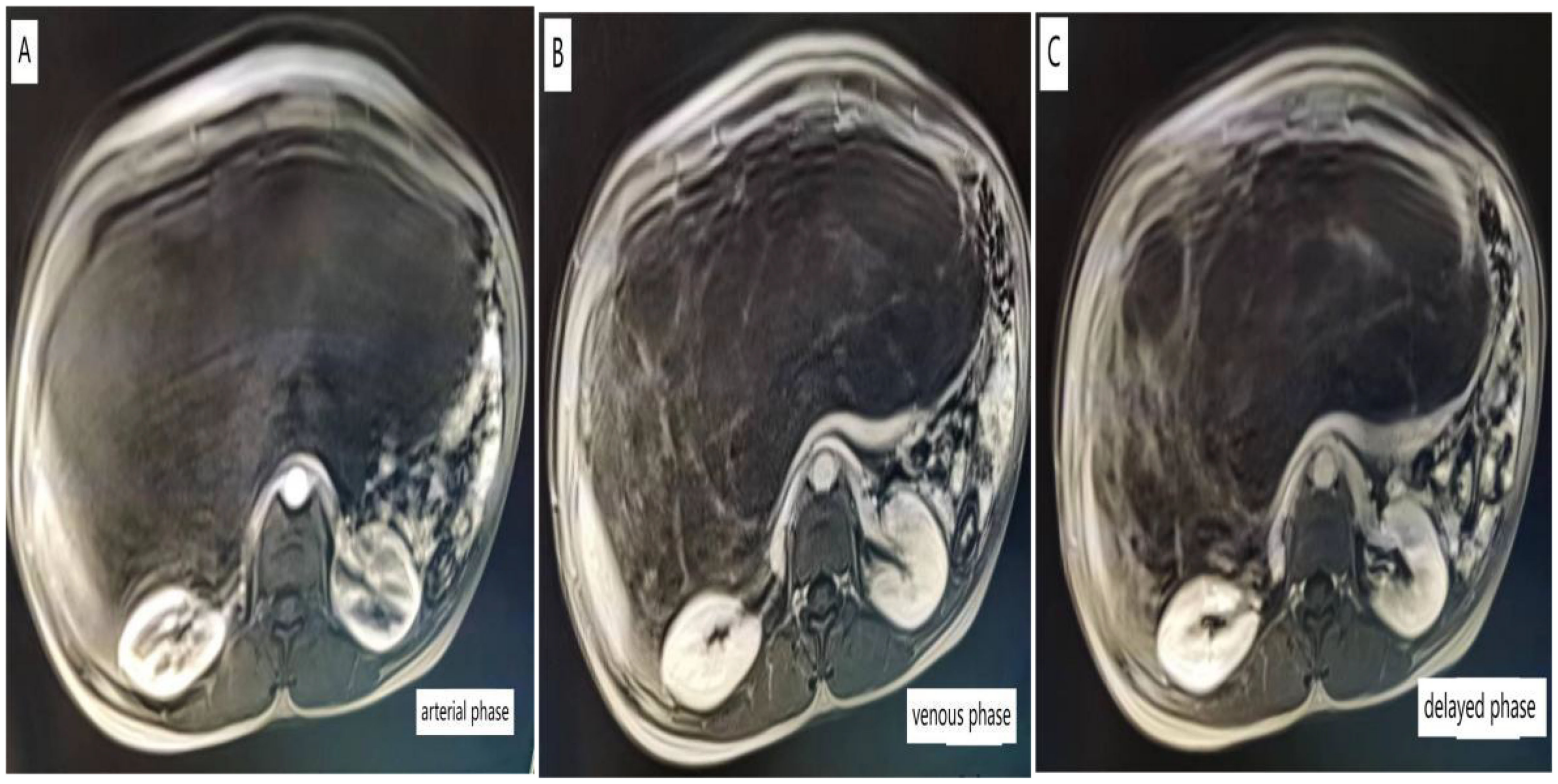
Pelvic-enhanced MRI images of arterial phase **(A)**, venous phase **(B)**, delayed phase **(C)**, and Pelvic MRI revealed huge lesions in the pelvis, which were cystic, uniform, and multiventricular, including multiple compartments.

Upon admission, relevant tests were completed: Tumor markers CA-125: 56.28 U/mL (reference value <35 U/mL), while other tumor markers were within normal ranges. Chest CT scan: A large cystic lesion was observed in the upper abdomen on the scan, with the largest dimension measuring approximately 9.8 × 6.3 cm; abdominal CT or MRI is recommended. Pelvic enhanced MRI shows a huge cystic lesion in the abdominal and pelvic cavities with multiple septations (some forming nodules), clear boundaries, measuring approximately 35.6 cm × 23.5 cm × 13.8 cm; enhancement scanning shows significant enhancement at the margins and septations; the lesion extends up to the T10 vertebral body and down to the pelvic floor, with surrounding structures significantly compressed and some septal nodules showing thickening (**Figure 1**).

**Figure 2:**
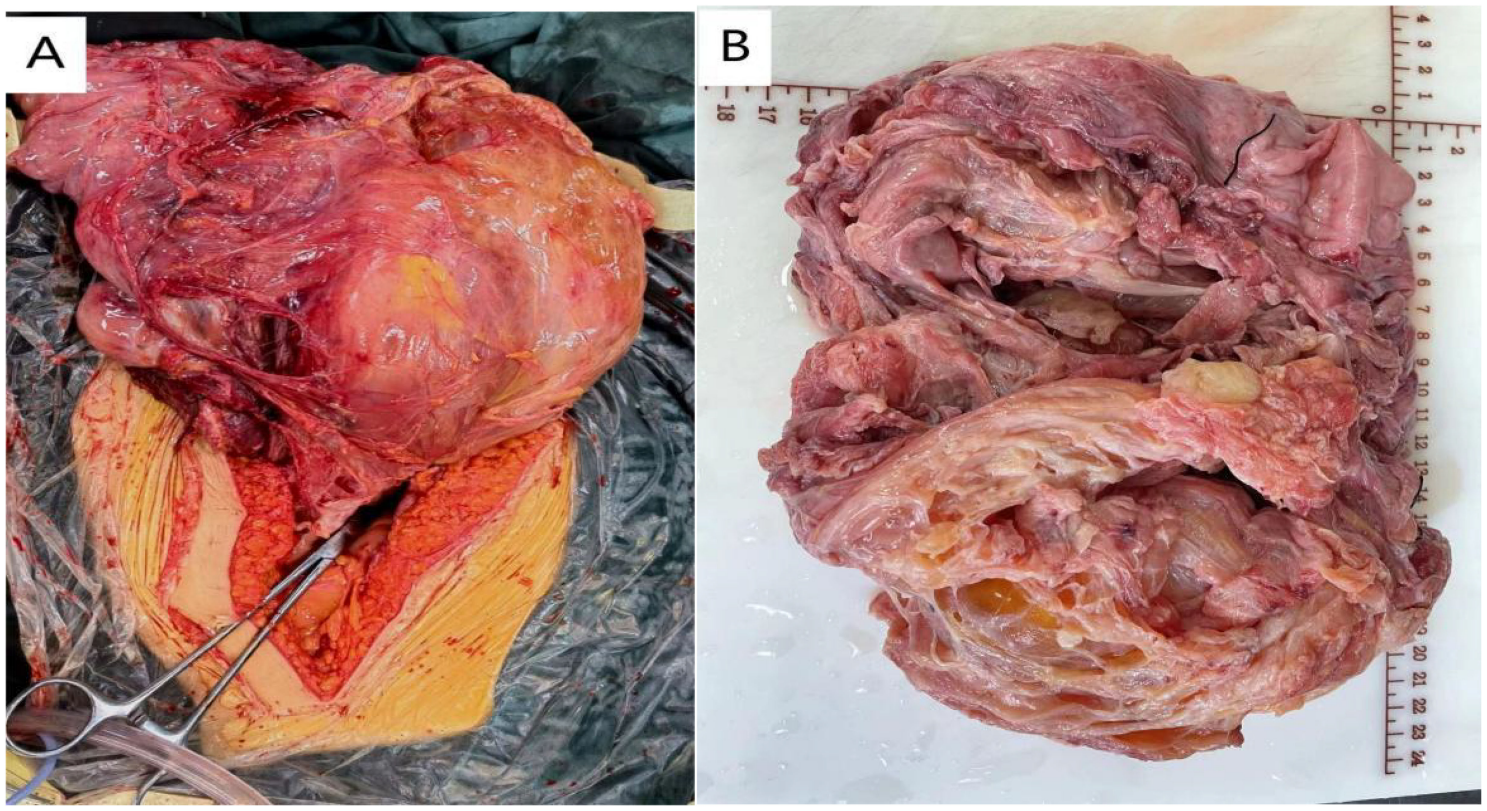
A large tumor occupies the pelvic cavity. The surface of the tumor is multilocular cystic, and the cystic fluid is pale yellow.

The patient had a history of pelvic cyst removal surgery 20 years ago, the pathological nature of which was unclear. Physical examination revealed no abnormalities in the cardiovascular and respiratory systems. Abdominal examination showed significant abdominal distension, with a huge tumor occupying the entire abdominal cavity. After ruling out contraindications for surgery, exploratory laparotomy revealed a huge cystic mass behind the peritoneum measuring approximately 36.0 cm × 24.0 cm × 14.0 cm, with a smooth surface, white, red, and pale yellow exudate, unclear borders, extensive adhesions to pelvic tissues, greater omentum, and transverse colon, connected to the right pelvic peritoneum, adjacent to blood vessels behind the right pelvic peritoneum, right ureter, and rectum, with unclear bladder involvement. The pelvic wall was densely adhered to the pubic symphysis, with extensive adhesions on the left, involving the left pelvic peritoneum. The wall of the huge pelvic mass was smooth, with the fluid resembling pale yellow jelly (**Figure 2**). Due to extensive adhesions between the tumor tissue and surrounding pelvic tissues, complete surgical resection appeared very difficult, but we decided to remove visible tumor tissue as much as possible.

**Figure 3:**
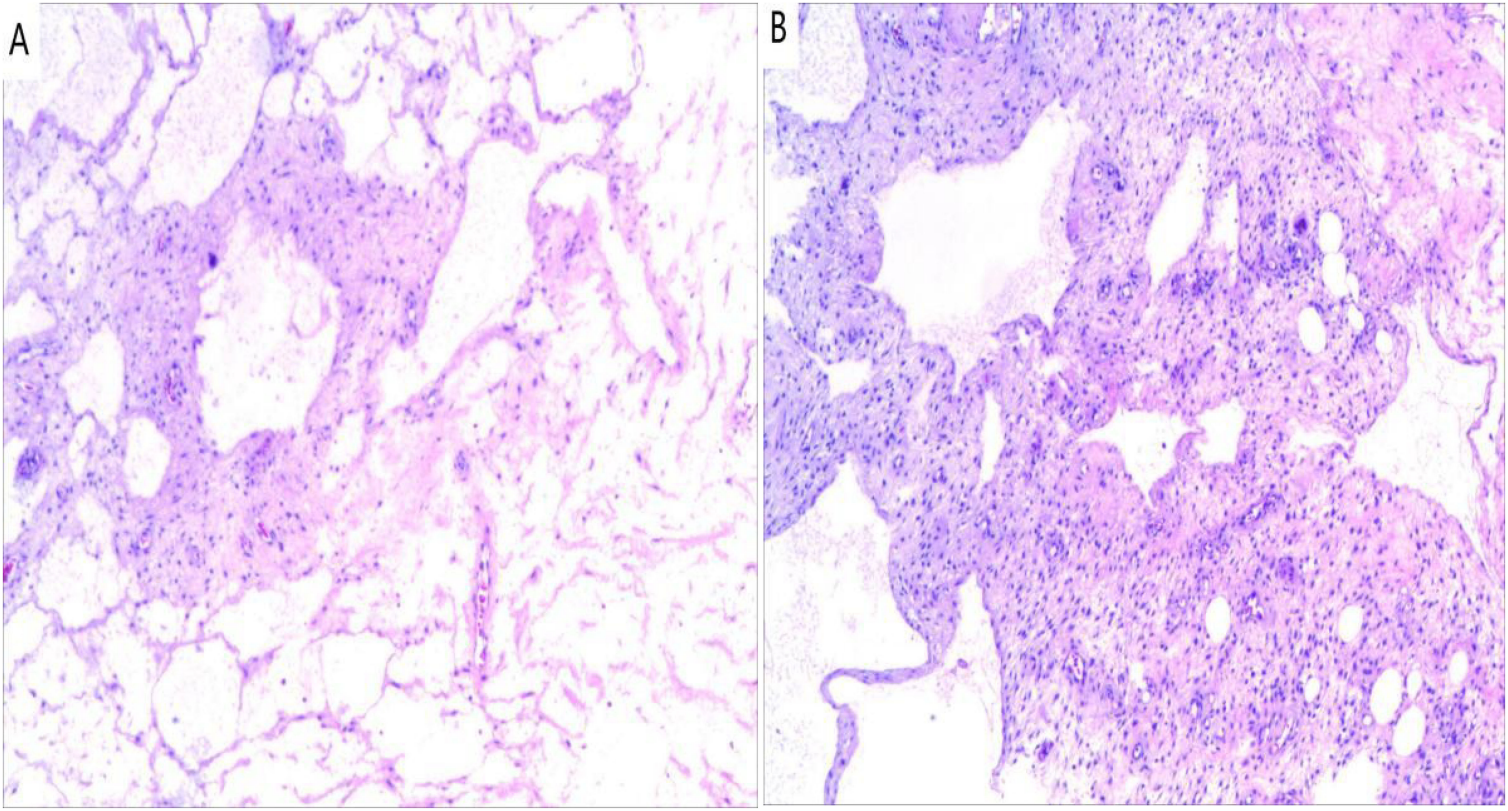
Histopathology (Hematoxylin and eosin). **(A)** Numerous dilatated lymphatic vessels (×100); **(B)** Lymphocytic aggregates in the cyst walls (×100).

Postoperative pathology revealed an “intra-abdominal mass” mesenchymal origin tumor with extensive cystic changes, combined with immunohistochemistry suggesting a tendency towards CL malformations. Immunohistochemistry results: (4th) inhibin α(-), desmin(+), CR (-), SMA (+), ki-67 (+1%), D2-40 (focal+), WT-1 (-), HMB-45 (-), CK5/6 (-), CD34 (-) (**Figure 3**).

The patient recovered well 8 days postoperatively and was able to eat normally. She was discharged on the 12th day postoperatively, recovering well with no pain or other complications. In the early postoperative period, it was recommended for the patient to consume easily digestible, high-nutrient protein foods and avoid high-fat, high-fiber, and difficult-to-digest foods. It was advised to undergo pelvic MRI every 3 months for 1 year postoperatively.

A pelvic MRI was performed during a 6 months follow-up, revealing a pelvic tumor measuring approximately 8.9 cm × 4.5 cm × 7.0 cm, with an irregular shape, unclear boundaries, and uneven internal echogenicity, Tumor recurrence was suspected. During the operation, the tumor was located behind the peritoneum and surrounded by large blood vessels. The tumor was large, adhered to the surrounding tissue, and had unclear boundaries. Therefore, the cause of tumor recurrence may be incomplete resection of tumor tissue. Reexamination found that the tumor was growing rapidly, and doctors at higher hospitals were asked to assist in treatment. Due to the specific location of the patient’s tumor and the history of multiple surgeries, the likelihood of complete resection is small if the patient undergoes surgical treatment again. On the other hand, the patient refused to open the abdomen again due to his own history of multiple operations, and chose to take traditional Chinese medicine. I will follow the patient for a long period of time.

## Discussion

Lymphatic malformation is a general term for abnormal lymphatic overgrowth. Currently, the main recognized cause is congenital malformations of the lymphatic system, which lead to abnormal expansion and proliferation of lymphatic vessels, resulting in lymphatic malformations (8). Trauma, partial lymphatic obstruction, and inflammation can also lead to secondary lymphatic malformations (9). CL is more common in the head, neck and armpit, and is more common in children, with no gender difference in incidence. Intraperitoneal morphology is seen in only 5% of cases. Retroperitoneal CL is particularly rare (4). Retroperitoneal CL is a rare condition and accounts for 1% of all CL (10). The main clinical manifestations were abdominal pain, abdominal mass, nausea and vomiting, and the rare manifestations were bleeding, rupture and torsion. Early diagnosis mainly depends on ultrasound, abdominal CT and pelvic MRI, and final diagnosis mainly depends on histopathology (11). Surgical excision is the primary treatment, and in our case, the patient was a middle-aged woman with a history of pelvic cyst surgery, and we suspected that her condition was secondary.

CT and MRI are the initial tests for the presence of abdominal masses (12). The clinical manifestations of retroperitoneal lymphatic CL are nonspecific and are associated with the size, location, and surrounding tissue of the tumor, which poses a challenge to diagnosis. CL should be distinguished from abdominal lymphoma, mesenteric cysts, secondary malignant tumors, pulmonary tuberculosis, and hydatid disease (13). The characteristics of pelvic CL were examined by various imaging methods. Ultrasound has a unique advantage in showing the size, location, content, and boundaries of cysts. However, when there is bleeding and necrosis in the capsule, the echo of the contents will produce some changes, affecting the judgment. Abdominal-enhanced CT is preferred. Abdominal-enhanced CT can observe tumor density, determine the relationship with surrounding tissues, blood vessels and organs, and distinguish retroperitoneal lymphangioma from intraperitoneal lymphangioma. MRI is more sensitive in showing bleeding and contents within cysts (14).

The diagnosis of pelvic CL ultimately requires pathological confirmation. Normal lymphatic vessels show proliferative endothelium mainly in the submucosal layer, with destruction of the muscle layer and serosa being characteristic for diagnosis. Immunohistochemically, D2-40 is a specific marker for lymphangioma, while CD31, CD34, von Willebrand factor, and VEGFR3 can also be helpful for diagnosis. By combining clinical presentation with these features, lymphangioma can be diagnosed more accurately (15).

Treatment depends on the nature of the symptoms, anatomical location, and potential complications of treatment, including systemic treatment, surgery, and radiation therapy (16). The patient reported in our case had extensive adhesions with the retroperitoneum and surrounding pelvic tissues, so we chose to surgically remove the entire tumor tissue. Surgical resection of the tumor is the preferred treatment for retroperitoneal lymphatic cysts. During surgery, attention should be paid to the relationship between the tumor and surrounding tissues, blood vessels, nerves, and organs to completely remove the tumor and reduce the rate of recurrence after surgery (17). Koshima I, et al. Lymphaticovenular anastomosis(LVA) combined sclerotherapy is a relatively less invasive approach that does not further aggravate or develop lymphedema, and LVA combined sclerotherapy can be used as an adjoint minimally invasive treatment option for CL. However, this type of treatment is very complex and challenging, requiring clear vascular recognition, which is not easy to achieve in large CL (18).

In our case, improve the following checks, such as liver function examination, kidney function examination, tumor markers, upper abdominal color ultrasound, gynecological color ultrasound examination and pelvic enhanced MRI, etc. An exploratory laparotomy was performed, and the postoperative pathology proved retroperitoneal CL. Pelvic MRI can be used as an effective means of postoperative follow-up examination. Due to the patient’s history of pelvic cystic tumor resection 20 years ago, the medical records of the patient are missing, so it is impossible to confirm whether the pathology 20 years ago diagnosed pelvic CL. When the patient was admitted to hospital, the tumor was huge and presented with abdominal distension, abdominal pain, constipation and other symptoms. Exploratory laparotomy was performed and the patient recovered well after surgery. However, we are thinking that there are still areas in our treatment that need to be improved. The reason for the recurrence of this patient is still unknown, and many experts consider whether the tumor is huge and completely adhered to the surrounding tissue, resulting in residual recurrence of tumor tissue, of course, this is only a guess, there is no actual evidence, and further research is needed. We can improve in the following aspects, for example, in the future operation, the contents of the cyst can be removed first, the tumor volume can be reduced, the difficulty of the operation can be reduced, and the operation time can be shortened, which can help patients recover better after surgery. Secondly, a single surgical resection may not achieve satisfactory results. For patients with large tumors and severe symptoms, a variety of treatment methods should be adopted, with surgical resection as the main treatment method and chemotherapy to treat residual lesions.

The aimed at this case study is to share our experience in the diagnosis and treatment of this rare giant cystic tumor in the retroperitoneum. Currently, there are well-established treatment plans for this disease. In the future, attention could be focused on further exploring CL relapse. Of course, this is just our one-sided opinion, and any inappropriate aspects should be criticized and corrected.

## Conclusion

Retroperitoneal cystic lymphatic malformations have a low incidence rate, and the diagnosis mainly relies on ultrasound, abdominal CT, pelvic MRI, etc. The final confirmation mainly depends on pathological histology, and surgical resection is the main treatment method. Long-term follow-up is required for this disease.

## Data Availability

The original contributions presented in the study are included in the article/supplementary material. Further inquiries can be directed to the corresponding author.
